# BacMet: antibacterial biocide and metal resistance genes database

**DOI:** 10.1093/nar/gkt1252

**Published:** 2013-12-03

**Authors:** Chandan Pal, Johan Bengtsson-Palme, Christopher Rensing, Erik Kristiansson, D. G. Joakim Larsson

**Affiliations:** ^1^Department of Infectious Diseases, Institute of Biomedicine, The Sahlgrenska Academy at the University of Gothenburg, Gothenburg, SE-413 46, Sweden, ^2^Department of Plant and Environmental Sciences, University of Copenhagen, Copenhagen, DK-1871, Denmark and ^3^Department of Mathematical Sciences, Chalmers University of Technology, SE-412 96, Gothenburg, Sweden

## Abstract

Antibiotic resistance has become a major human health concern due to widespread use, misuse and overuse of antibiotics. In addition to antibiotics, antibacterial biocides and metals can contribute to the development and maintenance of antibiotic resistance in bacterial communities through co-selection. Information on metal and biocide resistance genes, including their sequences and molecular functions, is, however, scattered. Here, we introduce BacMet (http://bacmet.biomedicine.gu.se)—a manually curated database of antibacterial biocide- and metal-resistance genes based on an in-depth review of the scientific literature. The BacMet database contains 470 experimentally verified resistance genes. In addition, the database also contains 25 477 potential resistance genes collected from public sequence repositories. All resistance genes in the BacMet database have been organized according to their molecular function and induced resistance phenotype.

## INTRODUCTION

Over the past decades, widespread use, misuse and overuse of antibiotics have accelerated the development of antibiotic resistance (1). The increasing ability of pathogenic bacteria to survive antibiotic therapy has thus been recognized as one of the largest threats to public health (2). A particular concern is that bacteria are becoming resistant to more than one antibiotic compound—so-called multidrug resistance. This is either due to co-selection of multiple resistance genes within a single bacterial cell (e.g. multiple plasmids or arrays of genes on a single plasmid) and/or due to the presence of resistance genes (e.g. efflux pumps) with a broad substrate range (3).

Biocides and metals have been used as antibacterial agents for centuries. For instance, copper (Cu) and silver (Ag) vessels have been used for maintaining quality in stored potable water (4). Various metal salts such as copper, magnesium (Mg), mercury (Hg), tellurium (Te), arsenic (As) and gold (Au) have been used for treating infectious diseases such as leprosy, tuberculosis, gonorrhoea and syphilis (5–9). More recently metals such as copper and silver have become widely used in household products, and disinfectants are regularly used in, for example, hospitals, industries and elsewhere to sanitize equipment (10–11). Copper, zinc and, to a lesser extent, cadmium (Cd) and arsenic are also used as animal growth promoters in certain regions, possibly mediated by their effects on the animal gut flora (12–15). In aquaculture and agriculture, practices of metal- and biocide-containing products in feed additives, organic and inorganic fertilizers, pesticides and antifouling products are also well known worldwide, resulting in enrichment of aquaculture sediments and river water with metals such as zinc, copper, cadmium and lead (Pb) (16–18). All such uses potentially create venues for biocide and metal resistance development, but also for increased antibiotic resistance as a side effect of bactericide usage. The EU Scientific Committee for Emerging and Newly Identified Health Risks recently concluded that exposure of bacteria to biocides and metals may boost the spread of antibiotic resistance (19–20). This is a parallel situation to the development of multidrug resistance, resulting from the presence of resistance genes for both antibiotics and biocides/metals located in close genetic vicinity of each other on mobile genetic elements, or by the presence of common resistance mechanisms with broader specificity (21–26).

There is, however, still a substantial knowledge gap and a lack of easily accessible organized data resources on biocide and metal resistance for exploring these sources of antibiotic resistance development and understanding the co- and cross-resistance mechanisms between antibiotics, biocides and metals. Currently, databases of antibiotic resistance genes exist, such as the Antibiotic Resistance Genes Database (27) and the Comprehensive Antibiotic Resistance Database (28). The Arthropod Pesticide Resistance Database (http://www.pesticideresistance.com/) and the Insecticide Resistance database (29) are examples of biocide resistance databases mainly focusing on genes in animals and plants. However, there are no specialized databases available for bacterial resistance genes to biocides or metals.

To facilitate research on co-selection, there is a need for a database on bacterial biocide- and metal-resistance genes with highly reliable content. With the growing use of biocides and metals as food preservatives, disinfectants and within antibacterial coatings, it is becoming increasingly important to understand mechanisms behind bacterial tolerance to such substances, regardless of any potential co-selection for antibiotic resistance. We have therefore created a manually curated collection of antibacterial biocide and metal resistance genes with experimentally confirmed function described in the scientific literature. Additionally, we have predicted antibacterial biocide and metal resistance genes based on sequence data available in different public repositories, such as NCBI GenBank (30), NCBI nonredundant protein database (31) and UniprotKB (32). The creation of BacMet—the Antibacterial Biocide and Metal Resistance Genes Database—is anticipated to facilitate research on single, co- and cross-resistance by enabling rapid screening of genomes and metagenomes. BacMet is freely available at http://bacmet.biomedicine.gu.se.

## DATABASE CONSTRUCTION AND CHARACTERISTICS

### Data collection

Only bacterial genes experimentally confirmed to be involved in resistance/tolerance were collected through an extensive literature search in PubMed (31) using a variety of terms relating to biocide and metal resistance. This was followed by searching the NCBI nonredundant protein database, UniprotKB and the Gene Ontology (33) database to identify the corresponding annotated biocide- and metal-resistance genes within these databanks, including references. Special attention was given to the listed references in each collected paper and the review papers in the biocide and metal resistance area to find primary articles describing yet other resistance genes. Each article was manually reviewed to identify the presence of targeted genes, any related genes, cross-resistance and experimental procedures and compounds used to verify the resistance phenotype. In a few cases, we found experimental evidence of resistance genes from the literature but could not include these genes in the database owing to lack of data submission to public databases.

A gene was considered to have experimentally confirmed resistance function only if (i) the removal/mutation or insertion/overexpression of a single gene of interest into the genome of a bacterial host, or into an inserted plasmid, resulted in an increased or decreased susceptibility to the biocide/metal, respectively; or (ii) insertion of a plasmid lacking the gene of interest showed increased susceptibility compared with insertion of the same plasmid carrying the gene. We have also included genes in operons, where the operon is experimentally confirmed to be involved in resistance, but where evidence for a resistance function of each individual component gene is lacking. The approach of identifying functional resistance genes (such as efflux pumps, chaperones and enzymes), by insertion, deletion or mutation of a particular target gene, has the potential to also identify regulators of such resistance genes. In many cases, such regulators are part of the same operon or resistance determinant gene cluster as the functional resistance gene, and are essential for resistance. In BacMet, to provide an overall picture of these resistance systems, we have included both types of genes with a note on whether they are regulators or functional resistance genes. Generally, the resistance phenotype conferred by regulators is highly dependent on expression level and context, thus a resistance phenotype should be not inferred only by their presence in a genome. Subsequently, metadata, such as source organism, location (plasmid/chromosomal) and gene description, were collected from NCBI GenBank and nonredundant protein database, UniprotKB and the Transporter Classification Database (34) and included into the database covering genes with experimentally confirmed function.

The scope of the database was to include resistance genes to metals as well as to antibacterial biocides. We have included genes providing resistance/tolerance to both essential metals (such as Fe, Mn, Cu, Co and Mg) and those that are only toxic (such as As, Cd and Hg), as all metals become toxic at elevated concentrations and thus can exert a selection pressure. Biocides were identified from a list of active substances for use in biocidal products on the EU market, i.e. European Commission Regulation (EC) No 1451/2007 (http://eur-lex.europa.eu/LexUriServ/site/en/oj/2007/l_325/l_32520071211en00030065.pdf). As this list is not complete, any compound for which we could find evidence for an intentional use to kill or prevent growth of bacteria (biocidal use) were classified as ‘biocides’. Classical antibiotic compounds were excluded, as resistance genes to antibiotics are covered in other databases such as the Antibiotic Resistance Genes Database and the Comprehensive Antibiotic Resistance Database; however, genes providing resistance to both biocides and antibiotics were included. The search for antibacterial biocide resistance genes also resulted in the identification of resistance genes to other chemical compounds that are toxic to bacteria, but are not generally used as biocides, such as dyes, organic solvents, etc. In principle, such compounds do also have the potential to co-select for antibiotic resistance, and we have therefore included genes conferring resistance to those compounds in the database, but under the heading ‘other compounds’.

### Curation of derived data

Initially, a core data set of 470 experimentally confirmed genes was created (referred to as ‘BacMet Experimentally Confirmed database') based on 421 articles from PubMed. The resistance genes in the experimentally confirmed database are biased toward model bacteria, since those are highly overrepresented in microbiology laboratory experiments (e.g. *Escherichia coli* and *Pseudomonas aeruginosa*). Thus, a large portion of resistance gene variants is almost certainly missing, as the resistance genes may differ between species and/or occur in different forms that are not (yet) experimentally investigated. Therefore, the experimentally confirmed data set of 470 resistance genes was used as a reference data set to obtain bacterial gene/protein sequences predicted to be conserved and have similar functions based on high sequence similarity. The predicted genes were selected from the NCBI nonredundant protein database by similarity searches using BLAST (version 2.2.25) (35), forming the larger ‘BacMet Predicted database'. Here, a multistage filtering was performed to collect homologous sequences using the reference data set. Initially, a fixed E-value cutoff of ≤0.01 (to obtain ‘significant' hits) was set, followed by a fixed coverage cutoff of 80% (to avoid inclusion of homologous sequences with good E-values solely due to virtually identical, but short, alignments between queries and target sequences) for all sequence matches. Thereafter, a 90% fixed identity cutoff was used for protein sequences originating from plasmids, while manual cutoffs (Supplementary Table S1) were determined for each individual chromosomal-borne sequence (as the chromosomal gene sequences vary substantially between species and occur in different forms). During the manual identity cutoff step, for every experimentally confirmed gene we accepted similar sequences down to an identity cutoff value where the gene/protein annotation was still relevant to the source experimental gene, including conserved hypothetical proteins. It should be noted that the derivation of appropriate identity cutoffs is a partially subjective decision, based on previous knowledge of sequence similarity within groups of proteins sharing the same function. Redundancy was removed by clustering all extracted homologous protein sequences with 100% identity cutoff using USEARCH (version 5.2.32) (36) to form the large database (referred to as ‘BacMet Predicted database’).

### Database contents

**Figure 1.**
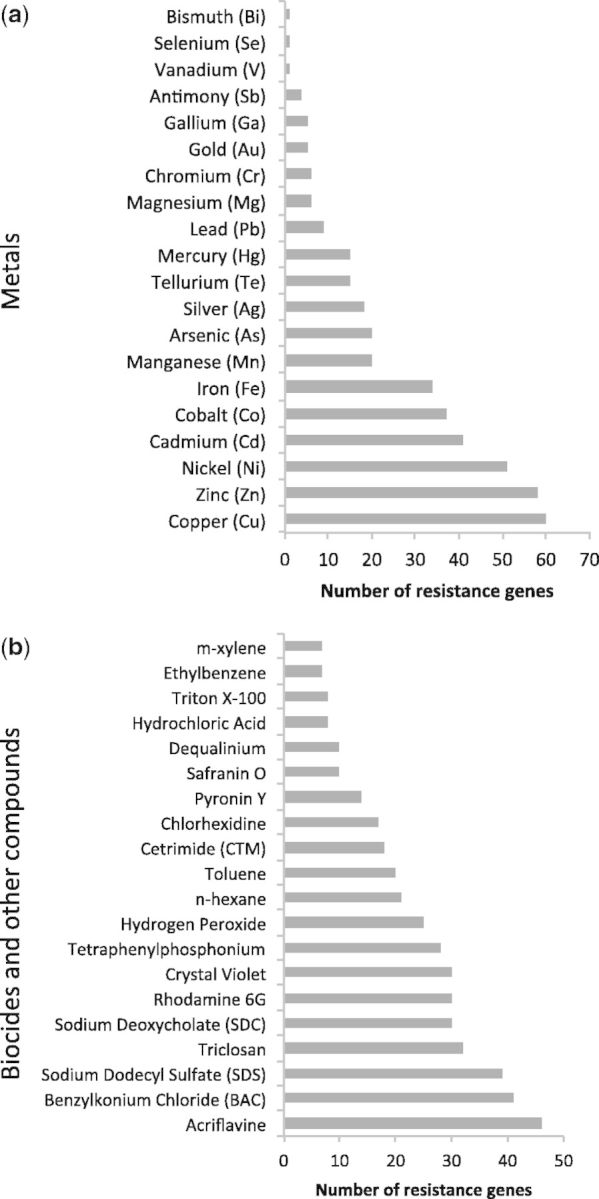
Summary of top 20 resistance genes for (**a**) metals and (**b**) biocides and other compounds in the experimentally confirmed database. Some of the included genes are represented in more than one category. The figure reflects the most well-studied compounds, although the actual substrate range is likely to be much broader for many genes.

**Figure 2.**
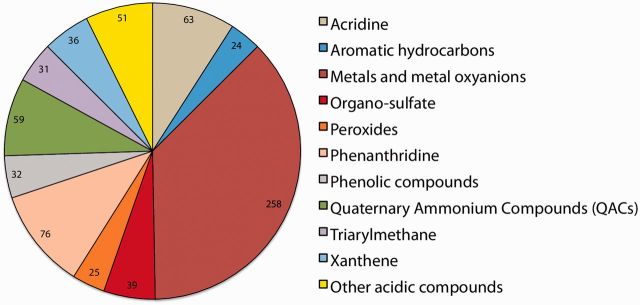
Top 10 chemical classes with corresponding resistance genes covered in the experimentally confirmed database. Some of the included genes are represented in more than one category due to a broad substrate range.

From 421 scientific articles from PubMed, we identified 470 bacterial genes experimentally confirmed to be involved in resistance/tolerance to biocides, metals and other similar chemical compounds. From these genes, 133 211 predicted sequences were extracted, generating a set of 25 477 nonredundant protein sequences included in the ‘BacMet Predicted database’ (Table 1). The 470 experimentally confirmed resistance genes listed in BacMet are shown to confer resistance to 84 different compounds. These include 20 metals, 41 biocides, i.e. chemicals used with the purpose of killing bacteria or preventing bacterial growth, and 23 ‘other chemical compounds’, verified as toxic to bacteria. The compounds have been listed by their names and classes in the BacMet database based on reviewed articles from PubMed, in which researchers used them to verify phenotypic changes governed by the presence of specific genes in laboratory experiments. Out of these 470 genes, 29 genes had a verified resistance phenotype for both biocides and metals. For metals, the highest number of resistance genes was found for copper (60 genes), followed by zinc (58 genes) and nickel (51 genes), whereas the biocide associated with most genes was acriflavine (46 genes) followed by benzylkonium chloride (BAC) (41 genes) and sodium dodecyl sulfate (39 genes). Summaries of the resistance gene findings are presented in Figures 1 and 2.

**Table 1.** General statistics of the BacMet database (October 2013)

Total experimentally verified resistance genes	470
Chromosome-borne genes in BacMet experimentally confirmed database	347
Plasmid-borne genes in BacMet experimentally confirmed database	123
Biocide resistance genes	212
Metal resistance genes	229
Genes with both biocide and metal resistance potential^a^	29
Total genes in BacMet predicted database (nonredundant)	25 477
Total compounds listed	84
PubMed references	421

^a^Not included in numbers given for biocide- and metal-resistance genes, respectively.

### Database schema

**Figure 3.**
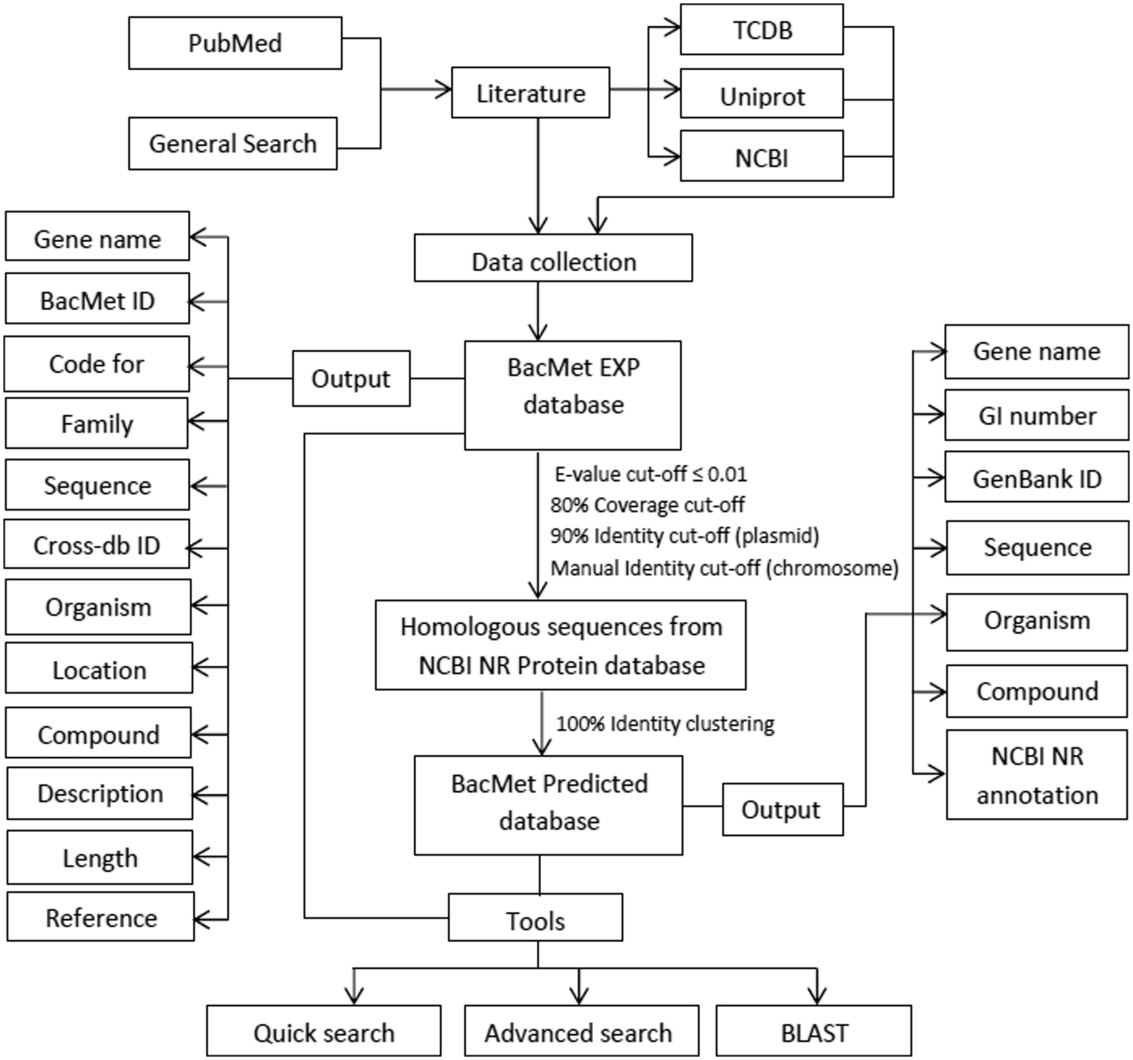
Database structure and workflow.

The BacMet workflow and architecture is described in Figure 3. The BacMet databases are built on Apache server 2.2.3 (http://www.apache.org). The database tables are stored in MySQL server 5.0.77 relational databases (http://www.mysql.com) and operated on RedHat Linux systems. The web interface was created in HTML using PHP, Java and Cascading style sheets. The Perl programming language (http://www.perl.org) along with the CGI, DBI and DBD modules were used for interconnection between the two databases, to retrieve the BacMet data from the MySQL databases, and to present result outputs in a web interface.

### Browsing and searching database

A tutorial has been included on the BacMet webpage, which describes database access, data extraction, browsing and searching the database, as well as how to submit data. The BacMet databases can be explored in the web interface through the ‘browse’ option, where one can browse resistance genes and chemical compounds. Resistance genes can be browsed by individual compounds, by the chemical classes they provide resistance to or by their name. Users can also browse all the chemical compounds in BacMet to obtain more information such as general uses and chemical classes along with an external link to a chemical database of biological interest such as ChEBI (37), ChEMBL (38) or PubChem (39). Alternatively, one may use the ‘search’ function to perform a quick search for any term in all fields in both databases separately, including, for example, gene names, names of biocides, chemical classes and names of metals. It is also possible to search specifically for e.g. plasmid or chromosomal genes or genes resistance to certain chemical classes using the ‘advanced search’ function.

The output for browsing and search results from the two databases is slightly different. For the predicted database output, most of the information is externally linked to the NCBI protein database, to allow the user to acquire more information of the genes depending on his or her interests. As the two databases are interconnected, users have the opportunity to obtain information on the source of every gene in the BacMet predicted database from the experimentally confirmed database. Alternatively, users also can obtain all information on predicted genes with similar names by a single click on experimental confirmed genes.

### BLAST search engine

A BLAST search web interface has been implemented to query the BacMet databases. A modified stand-alone version of the BLAST program (NCBI wwwblast version 2.2.2) (31) has been implemented on the BacMet web server for similarity searches against the BacMet sequence databases. The BLAST query can be performed against both BacMet databases using nucleotide and protein sequences as input. BLAST hit records can be used to obtain more information about the matched sequences in the BacMet database.

### Data download

All the data included in the BacMet databases are available to download for offline analysis. All the data files, including protein sequences in FASTA format, can be downloaded in compressed form (.zip format), or the user can choose to download individual files as required. Along with the data from the BacMet database, users can also download in-house developed software to analyze genomes and metagenomes for screening biocide and metal resistance genes locally.

### Data submission

A web-interface has been included for users to submit their data on resistance genes that are not listed in the BacMet database, along with the information of experimental support in the scientific literature. The submitted data will be added to the BacMet database after manual curation.

## DISCUSSION AND CONCLUSION

We here present a collection of antibacterial biocide and metal resistance genes that have experimental evidence, along with a larger set of genes with computationally predicted resistance function. To our knowledge, this is the first comprehensive resource of antibacterial biocide and metal resistance genes. Along with this, we provide in-house-developed software to analyse genomes and metagenomes locally, to enable screening for biocide- and metal-resistance genes in large-scale sequence data sets.

Although we have performed an extensive search to extract as many experimentally confirmed resistance genes as possible, we believe that we may still have missed some genes. Therefore, we encourage researchers to submit their data on experimentally confirmed resistance genes function, along with all related information. We also believe, due to the cross-resistance characteristic, some resistance genes in BacMet have the potential to confer resistance to various other biocide and metal compounds that have not been experimentally verified yet. Therefore, the list of compounds in BacMet, and every individual gene resistant toward these compounds, will be expanded when new experimental data are available. Importantly, phenotypic resistance to antibacterial biocides is described for a much larger range of compounds than those included in the database (40–42). This suggests that there are many more resistance genes to be discovered. We plan to update the BacMet database continuously with newly available experimental work and novel resistance gene information from the scientific literature and data submitted by users.

We believe that BacMet will facilitate research to understand co- and cross-resistance of biocide and metals to antibiotics within bacterial genomes, as well as in complex microbial communities (metagenomes) from different environments. The database will also facilitate gene annotation of new genomes and metagenomes. All in all, this may help us to better understand the bacterial resistome. Because biocides and metals are nowadays widely used as food preservatives, disinfectants and on antibacterial coatings for medical devices and health care facilities, as well as in residential and industrial settings, tolerance to these compounds by bacteria is a growing and upcoming concern as well. Hence, this database will not only allow microbiologists and toxicologists to address antibiotic resistance development in a more comprehensive way in the future, but it also may constitute an important resource for manufacturers of metal surfaces and coatings, biocide manufacturing companies and producers of food preservatives to understand the development of tolerance mechanisms to their products and possibly manage the use of metals and biocides in a more sustainable way.

## SUPPLEMENTARY DATA

Supplementary Data are available at NAR Online.

## FUNDING

Swedish Research Council FORMAS [2012-86]; Swedish Research Council VR; Gothenburg Centre for Marine Research (to D.G.J.L.). Funding for open access charge: Swedish Research Council FORMAS [2012-86].

*Conflict of interest statement*. None declared.

## References

[1] Wright GD (2010). The antibiotic resistome. Expert Opin. Drug Discov..

[3] Nikaido H (2009). Multidrug resistance in bacteria. Annu. Rev. Biochem..

[4] Alexander JW (2009). History of the medical use of silver. Surg. Infect. (Larchmt).

[5] Frazer AD, Edin MB (1930). Tellurium in the treatment of syphillis. Lancet.

[6] Keyes EL (1920). The treatment of gonorrhea of the male urethra. JAMA.

[7] Hodges NDC (1889). The value of mercuric chloride as a disinfectant. Science.

[8] Pereira J (1836). Materia medica, or pharmacology, and general therapeutics. Lond. Med. Gaz..

[9] Kayne GG (1935). The use of sanocrysin in the treatment of pulmonary tuberculosis: (Section of Medicine). Proc. R. Soc. Med..

[10] McDonnell G, Russell AD (1999). Antiseptics and disinfectants: activity, action, and resistance. Clin. Microbiol. Rev..

[11] Russell AD (2003). Biocide use and antibiotic resistance: the relevance of laboratory findings to clinical and environmental situations. Lancet Infect. Dis..

[12] Castillo M, Martin-Orue SM, Taylor-Pickard JA, Perez JF, Gasa J (2008). Use of mannanoligosaccharides and zinc chelate as growth promoters and diarrhea preventative in weaning pigs: effects on microbiota and gut function. J. Anim. Sci..

[13] Li YX, Xiong X, Lin CY, Zhang FS, Wei L, Wei H (2010). Cadmium in animal production and its potential hazard on Beijing and Fuxin farmlands. J. Hazard. Mater..

[14] Lucas IAM, Livingstone RM, McDonald I (1961). Copper sulphate as a growth stimulant for pigs: effect of level and purity. Anim. Prod..

[15] Nachman KE, Baron PA, Raber G, Francesconi KA, Navas-Acien A, Love DC (2013). Roxarsone, inorganic arsenic, and other arsenic species in chicken: a U.S.-based market basket sample. Environ. Health Perspect..

[16] Burridge L, Weis JS, Cabello F, Pizarro J, Bostick K (2010). Chemical use in salmon aquaculture: a review of current practices and possible environmental effects. Aquaculture.

[17] Dean RJ, Shimmield TM, Black KD (2007). Copper, zinc and cadmium in marine cage fish farm sediments: an extensive survey. Environ. Pollut..

[18] Mendiguchía C, Moreno C, Mánuel-Vez MP, García-Vargas M (2006). Preliminary investigation on the enrichment of heavy metals in marine sediments originated from intensive aquaculture effluents. Aquaculture.

[19] SCENIHR (Scientific Committee on Emerging and Newly Identified Health Risks) (2009). Assessment of the Antibiotic Resistance Effects of Biocides.

[20] SCENIHR (Scientific Committee on Emerging and Newly Identified Health Risks) (2010). Research Strategy to Address the Knowledge Gaps on the Antimicrobial Resistance Effects of Biocides.

[21] Baker-Austin C, Wright MS, Stepanauskas R, McArthur JV (2006). Co-selection of antibiotic and metal resistance. Trends Microbiol..

[22] Nies DH (2003). Efflux-mediated heavy metal resistance in prokaryotes. FEMS Microbiol. Rev..

[23] Levy SB (2002). Active efflux, a common mechanism for biocide and antibiotic resistance. J. Appl. Microbiol..

[24] Chuanchuen R, Beinlich K, Hoang TT, Becher A, Karkhoff-Schweizer RR, Schweizer HP (2001). Cross-resistance between triclosan and antibiotics in *Pseudomonas aeruginosa* is mediated by multidrug efflux pumps: exposure of a susceptible mutant strain to triclosan selects nfxB mutants overexpressing MexCD-OprJ. Antimicrob. Agents Chemother..

[25] Cavaco LM, Hasman H, Aarestrup FM (2011). Zinc resistance of *Staphylococcus aureus* of animal origin is strongly associated with methicillin resistance. Vet. Microbiol..

[26] Hasman H, Aarestrup FM (2005). Relationship between copper, glycopeptide, and macrolide resistance among *Enterococcus faecium* strains isolated from pigs in Denmark between 1997 and 2003. Antimicrob. Agents Chemother..

[27] Liu B, Pop M (2009). ARDB—Antibiotic resistance genes database. Nucleic Acids Res..

[28] McArthur AG, Waglechner N, Nizam F, Yan A, Azad MA, Baylay AJ, Bhullar K, Canova MJ, De Pascale G, Ejim L (2013). The comprehensive antibiotic resistance database. Antimicrob. Agents Chemother..

[29] Megy K, Emrich SJ, Lawson D, Campbell D, Dialynas E, Hughes DS, Koscielny G, Louis C, Maccallum RM, Redmond SN (2012). VectorBase: improvements to a bioinformatics resource for invertebrate vector genomics. Nucleic Acids Res..

[30] Benson DA, Cavanaugh M, Clark K, Karsch-Mizrachi I, Lipman DJ, Ostell J, Sayers EW (2013). GenBank. Nucleic Acids Res..

[31] NCBI Resource Coordinators (2013). Database resources of the national center for biotechnology information. Nucleic Acids Res..

[32] Uniprot Consortium (2013). Update on activities at the Universal Protein Resource (UniProt) in 2013. Nucleic Acids Res..

[33] Blake JA, Dolan M, Drabkin H, Hill DP, Li N, Sitnikov D, Bridges S, Burgess S, Buza T, McCarthy F (2013). Gene ontology annotations and resources. Nucleic Acids Res..

[34] Saier MH, Yen MR, Noto K, Tamang DG, Elkan C (2009). The transporter classification database: recent advances. Nucleic Acids Res..

[35] Boratyn GM, Camacho C, Cooper PS, Coulouris G, Fong A, Ma N, Madden TL, Matten WT, McGinnis SD, Merezhuk Y (2013). BLAST: a more efficient report with usability improvements. Nucleic Acids Res..

[36] Edgar RC (2010). Search and clustering orders of magnitude faster than BLAST. Bioinformatics.

[37] Hastings J, de Matos P, Dekker A, Ennis M, Harsha B, Kale N, Muthukrishnan V, Owen G, Turner S, Williams M (2013). The ChEBI reference database and ontology for biologically relevant chemistry: enhancements for 2013. Nucleic Acids Res..

[38] Gaulton A, Bellis LJ, Bento AP, Chambers J, Davies M, Hersey A, Light Y, McGlinchey S, Michalovich D, Al-Lazikani B (2012). ChEMBL: a large-scale bioactivity database for drug discovery. Nucleic Acids Res..

[39] Bolton EE, Wang Y, Thiessen PA, Bryant SH (2008). PubChem: integrated platform of small molecules and biological activities. Annu. Rep. Comput. Chem..

[40] Walsh SE, Maillard JY, Russell AD, Catrenich CE, Charbonneau DL, Bartolo RG (2003). Development of bacterial resistance to several biocides and effects on antibiotic susceptibility. J. Hospital Infect..

[41] Arioli S, Elli M, Ricci G, Mora D (2013). Assessment of the susceptibility of lactic acid bacteria to biocides. Int. J. Food Microbiol..

[42] Fraise AP (2002). Susceptibility of antibiotic-resistant cocci to biocides. J. Appl. Microbiol..

